# Anxiety in depression

**DOI:** 10.1192/j.eurpsy.2021.1831

**Published:** 2021-08-13

**Authors:** E. Tahmazov, G. Robert, M. Walter, C. Lemey

**Affiliations:** 1 Urci Mental Health Department, Brest Medical University Hospital, Brest, France; 2 Ea 7479 Spurbo, University of Western Brittany, Brest, France; 3 Academic Department Of Adult Psychiatry, Guillaume Régnier Hospital, Ea 4712, Rennes University Hospital, Rennes, France; 4 Centre Research Unit Ea 4712 Behavior And Basal Ganglia, Rennes University Hospital, Rennes, France

**Keywords:** Anxiety, Depression, anxious depression

## Abstract

**Introduction:**

There are different clinical forms combining anxiety and depression and it is essential to identify them because they will require different management. Among these clinical forms, there is that including anxiety as a symptom within the depressive episode: the anxious depression.

**Objectives:**

The objective is to find the characteristics of this anxious depression.

**Methods:**

We conducted a literature review on the PubMed® site giving access to the MEDLINE® database, as well as on the Google Scholar® search engine and retained 127 articles.

**Results:**

By studying anxiety as a symptom of the depression, we identify on the pathophysiological level different neurobiological mechanisms (neuroanatomical, biological, immunological and endocrinological) involved in types of symptoms of different anxiety. Thus, by adopting a dimensional point of view, we can say that there are various anxiety symptoms which can be included in multiple forms of anxiety within the depression: psychic anxiety (anxiety and irritability), somatic anxiety (hypochondria, sweating, cardiological, respiratory, gastrointestinal and urinary symptoms), motor anxiety (agitation), anxious arousal (somatic anxiety, fear, panic) or anxious apprehension (anticipatory anxiety and worry). The prognosis which emerges from it is of a more pejorative evolution, and has specificities on which an increased attention is required, such as suicidal behavior which is more frequently described for example. The treatment must be psychotherapeutic, sociotherapeutic, and medication by antidepressant treatment, with SSRIs in the first line.

**Conclusions:**

It is therefore essential to identify the clinical presentation of the anxious depression because it has specific semiological, neurobiological, prognostic and therapeutic characteristics.

**Disclosure:**

No significant relationships.

